# Editorial: Recent advancements in AI-assisted gynecologic cancer detection

**DOI:** 10.3389/fonc.2026.1920582

**Published:** 2026-08-11

**Authors:** Imran Ashraf, Isabel de la Torre Diez, Jin-Ghoo Choi

**Affiliations:** 1 School of Computer Science and Engineering, Yeungnam University, Gyeongsan-si, Republic of Korea; 2 Department of Signal Theory, Communications and Telematics Engineering, University of Valladolid, Valladolid, Spain

**Keywords:** AI-assisted imaging, feature fusion, gynecologic cancer, machine learning, precision cancer detection

Cervical, ovarian, endometrial, vulvar, and vaginal are among gynecologic cancers that pose significant threat to women’s health globally. Recent advancements in medical sciences have helped diagnose cancer precisely, making timely treatment possible. However, early detection of gynecologic cancers is difficult because of heterogeneous imaging modalities, variations in color, noise, etc. In addition, treating gynecological cancers requires personalized treatment. In this regard, Artificial Intelligence (AI) holds paramount importance to get more accurate diagnoses and streamline clinical workflows thereby enhancing patient outcomes. Integrated with gynecological oncology, AI can help in the diagnosis and prognosis of gynecological cancer and dealing with malignancies affecting the female reproductive system. The Research Topic, “Recent advancements in AI-assisted gynecologic cancer detection” was initiated to cover and highlight recent advancements in AI in gynecological oncology, to achieve early detection of cancer, risk identification of various type of cancers, and personalized treatment plans for better patient outcomes.

This Research Topic primarily emphasizes on AI-assisted detection, however, the term is used in a broad clinical context. Detection in this context encompasses the full continuum of cancer care, including early diagnosis, risk stratification, prognostic prediction, treatment planning, etc. AI applications in gynecologic oncology are integrative, where detection, prognosis, and intervention are increasingly interconnected.

The studies in this Research Topic comprises works that survey, quantify, and synthesize the broader AI landscape in the field of gynecologic oncology. A comprehensive synthesis of AI is provided by Dina et al., along with the use of radiomics, and multiomics applications in ovarian cancer diagnosis. Two prominent theses in the review are multimodal data integration, and the recognition that interpretable AI approaches are a necessary enhancement for clinical deployment. Complementing this review is the work of Tulimil et al. that offers a comprehensive review on using AI for cervical cancer care, involving screening, diagnosis, prognosis, and treatment planning. The study acknowledges the need for multi-center collaboration, and lack of high-quality datasets.

Bridging synthesis and quantification, the study maps cervical cancer screening research studies published between 2000 to 2024. It indicates a substantial increase in publications, identifying AI as one of the most rapidly accelerating trends in published work. The bibliometric analysis further shows the disparities in global research trends, thereby indicating that high-income countries dominate in research compared to low- and middle-income countries. Zheng et al. provide a meta-analysis offering a quantitative synthesis of AI-based MRI performance in endometrial cancer. Strong diagnostic performance is confirmed with a pooled AUC of 0.90 for cervical stroma. In addition, QUADAS-2 quality assessment and Deeks’ funnel plot for publication bias further strengthens methodological transparency.

Collectively, a higher number of studies in the current Research Topic focus on automated diagnosis and prognosis, thereby indicating the potential and use of AI approaches. These studies demonstrate that the field of automated diagnosis is progressing from simple single-modality classifiers to sophisticated, multimodal, and architecturally innovative systems that are capable of supporting real-world clinical workflows. Kim et al. contributes with automated vascular segmentation for gynecologic oncology, using the nnU-Net v2 framework, achieving a Dice similarity coefficient of approximately 0.952 for major pelvic vessels. The accurate preoperative mapping of pelvic vasculature is an important contribution which is essential for surgical safety in radical hysterectomy and lymphadenectomy. The study’s. complementary approach in the domain of risk prediction, indicates that interpretable ML using accessible reproductive and metabolic markers, can deliver clinically useful uterine fibroid risk stratification. This approaches reduces the need for expensive imaging or molecular testing. The random forest model achieves an AUC of 0.734, which is moderate yet clinically meaningful in a primary care screening context. From the transparency point-of-view, the integration of SHAP-based explainability, illustrates how interpretability bridges the gap between algorithmic performance and clinical trust.

The complementary potential of transfer learning and federated learning (FL) is explored by Guo et al., and Li et al.. Guo et al. demonstrates the potential of multi-sequence deep transfer learning to predict endometrial cancer. The combined clinical-DTL model achieves an impressive AUC of 0.972 by integrating T2WI, ADC, and CE-T1WI sequences with independent clinical predictors, demonstrating the potential of multimodal fusion over single-modality approaches. Li et al. address a dimension largely absent from the current AI-in-medicine literature which is fairness and equity of FL frameworks across multi-institutional collaborations. The FedCMC model introduces a dual data-and-model contribution assessment mechanism that incentivizes high-quality institutional participation while maintaining prediction accuracy (AUC 0.83-0.90 across four centers). The work provides a technical contribution, as well as, a governance model where it demonstrates how AI development can be structured for smaller or lower-resource institutions to obtain advantages.

The contrastive learning framework by Zhang et al. further advances multimodal integration. The framework incorporates modalities like CT, ultrasound, and clinical data for preoperative lymph node metastasis prediction in cervical cancer, reporting remarkable 92.31% accuracy and an AUC of 0.88. The framework’s 80.0% sensitivity, while promising, signals that a meaningful proportion of true metastases would be missed, a clinically important consideration given that undetected nodal disease can fundamentally alter surgical and systemic treatment decisions. Wang et al. demonstrate that that a DL system trained on ultrasound images, can achieve AUC scores between 0.811 and 0.858 across training, validation, and testing cohorts for endometrial cancer detection. The study’s explicit goal of empowering less experienced physicians in primary care settings is clinically significant. The study presents the AI framework not as a tool for specialist augmentation but as a mechanism for extending diagnostic capability of inexperienced physicians. The focus of the framework which is under-resourced frontline experts, deserves substantially more research investment.

Despite a large number of studies in gynecologic cancer, several subtypes are still under investigated. Several contributions focus specifically on rare gynecologic malignancies and advanced imaging paradigms. For example, Zhang et al. address an important topic in gynecologic oncology, i.e., mucinous ovarian carcinoma, which is rather rare. With a modest sample size of 80 patients, the graph neural network provides a C-index of 0.8254 which is superior to established clinical factors like FIGO. The GNNExplainer is used to provide interpretability which further strengthens clinical trust by pointing out histopathological features. Mucinous ovarian carcinoma being rare cancer subtype, larger multi-institutional validation is essential before integration of such tools in pathology workflows. Yang et al. make a conceptually important contribution by demonstrating that habitat-based radiomics, which partitions the tumor into biologically distinct sub-regions using k-means clustering on voxel intensity and entropy, systematically outperforms conventional whole-tumor radiomics for predicting parametrial invasion in early-stage cervical cancer. The habitat 3 model achieves a near-perfect training AUC of 1.00, with a meaningful but expected drop to 0.85 on the test cohort with a sample of 110 patients. More importantly, the habitat framework offers a theoretically grounded mechanism linking imaging phenotype to tumor microenvironment biology

Two case reports in this Research Topic address rare gynecologic entities from a diagnostic perspective, offering complementary value to the algorithmic studies. Wang et al. provide a detailed sonographic characterization of serous surface papillary borderline ovarian tumors (SSPBOTs), a subtype that frequently mimics malignant germ cell tumors on standard ultrasonography. The detailed sonographic description is provided, especially the “microcystic sign” and ‘irework-like” perfusion pattern, to provide diagnostic markers for better classification. The successful fertility-sparing approach in a 22-year-old patient is discussed to show the clinical significance of accurate diagnosis. Nobile et al. document an exceptionally rare synchronous presentation of mammary-like vulvar adenocarcinoma and primary breast carcinoma, illustrating the diagnostic complexity that arises when standard classification frameworks encounter entities that straddle conventional anatomical and histological boundaries. The focus is on distinguishing dual primaries from metastatic spread. The adopted process of combining imaging, histopathology, and immunohistochemistry, is commendable such ambiguous and rare presentations. Taken together, these cases reinforce a broader message of the Research Topic: rare entities require both technological innovation and structured clinical expertise, and neither alone is sufficient.

Considered collectively, the studies in this Research Topic provide a coherent and consequential picture about the transition of AI in gynecologic oncology from proof-of-concept algorithms to systems aiming for practical clinical deployment. Performance benchmarks that would have been considered exceptional five years ago, AUC values above 0.90, segmentation accuracy rivalling subspecialist radiologists, risk stratification models outperforming established clinical staging systems, are now reported with increasing regularity across diverse malignancies and clinical tasks. This trajectory is real, and it is accelerating. At the same time, the gap that separates technical performance from clinical implementation is also explored. First, external validation remains the most consistently cited limitation across the Research Topic. Studies rely on a smaller datasets, lack manual labeling, use data from single institutes, or with the same settings in different institutes, and models lack generalizability. No algorithm, regardless of internal performance, can be responsibly deployed in routine clinical practice without demonstrated generalizability across diverse patient populations, imaging equipment, etc. Such issues necessitate generalizable models tested on data from heterogenous institutes with diverse populations. Second, explainable AI has evolved from a desirable feature to a clinical necessity. It is demonstrated that explainability is not simply an ethical requirement but a practical tool that enhances experts trust on AI models. Third, multimodal data integration is emerging as the dominant technical direction across the field. Single-model are being superseded by frameworks that fuse imaging sequences, clinical variables, genomic profiles, and pathological features to get more accurate results with higher precision. The studies in this Research Topic collectively suggest that the most impactful near-term research should be in prospective multicenter data collection, standardized benchmarking protocols, regulatory science for AI medical devices, and clinician education on AI tool interpretation and appropriate use.

